# Management of Open Abdomen: Single Center Experience

**DOI:** 10.1155/2013/584378

**Published:** 2013-11-17

**Authors:** Hakan Yanar, Emre Sivrikoz

**Affiliations:** Department of General Surgery, Istanbul School of Medicine, Istanbul University, Millet Cad., Fatih, 34390 Istanbul, Turkey

## Abstract

*Aim*. The authors reviewed their experience in the management of open abdomen using the vacuum-assisted closure (VAC), in order to assess its morbidity, and the outcome of abdominal wall integrity. *Methods*. A retrospective review was performed using the trauma registry to identify patients undergoing temporary abdominal closure (TAC) either using Bogota Bag (BB) or VAC, from January 2006 to December 2012. Inclusion criteria were TAC and survival to definitive abdominal closure. Data collected included age, indication for TAC, number of operating room procedures, primary fascial closure rate, and complications. *Results*. During the study period, 156 patients required one type of TAC. Mean number of operations required in BB group was 3.04 as compared to 1.96 in VAC group (*P* = 0.006). Survival was significantly increased in the VAC group (*P* < 0.001). The difference in primary closure rates did not reach statistical significance (25% vs. 55%; *P* = 0.074). Complications were observed less frequently in the VAC group (*P* = 0.047). The mean time for fascial closure was 21 (±12) days in the BB group, as opposed to 6 (±3) days in the VAC group (*P* < 0.001). *Conclusion*. The vacuum assisted closure (VAC) has a significantly faster rate of closure, requires less number of operations, and is associated with a lower complication rate.

## 1. Introduction

 Damage control surgery has evolved two decades ago as a life-saving adjunct in the management of severe trauma requiring immediate surgical intervention. The concept was first described in 1993 by Rotondo et al. as “a promising alternative” to definitive laparotomy in exsanguinating patients with major vascular and multiple visceral penetrating abdominal injuries [1]. Subsequently, it has been shown that initiating damage control early on before the patient reaches the extremis (massive blood loss (>10 packs RBC), severe trauma (ISS > 25), hypothermia (<34°C), acidosis (pH < 7.25), and coagulopathy (aPTT > 19 sec)) reduces mortality [2].

 When damage control surgery is applied in an attempt to increase survival, surgeons are faced with a secondary problem, namely, the abdominal compartment syndrome (ACS). Early applications of damage control approach were concluded with abbreviated abdominal closure (quick running suture and towel clips). After recognition of the morbidity and mortality attributed to ACS, several methods were developed to avoid this complication [3]. The application of Bogota Bag (sterile serum bags) became the most popular and effective method of temporary abdominal closure.

 A decade ago, the concept of applying negative pressure was introduced by Barker et al. to promote primary fascial closure [4]. Following the introduction of the vacuum-pack technique, a more comprehensive method to deliver negative pressure therapy to an open abdominal wound was developed—the vacuum assisted closure (VAC) therapy. This technique was shown to enable late fascial closure in open abdomen patients up to a month after initial laparotomy [5]. 

 We aimed to review our experience in the management of “traumatic or nontraumatic open abdomen” as our practice transitioned from the Bogota Bag to the vacuum-assisted closure (VAC) in order to assess the morbidity and the outcome of abdominal wall integrity of both techniques.

## 2. Materials and Methods

A retrospective review was performed using the trauma registry to identify the patients undergoing temporary abdominal closure (TAC) either using Bogota Bag (BB) or VAC, from January 2006 to December 2012. Inclusion criteria were TAC and survival to definitive abdominal closure. Data collected included age, indication for TAC, number of operating room procedures, primary fascial closure rate, and complications. Complications were defined as intraabdominal infections, dehiscence, and hernia. Indication for TAC included hollow viscus perforation, anastomotic leakage, abdominal compartment syndrome, and damage control surgery.

The demographics of the two study groups were compared using Pearson's chi square or Fisher's exact test as appropriate for categorical variables, and student's *t*-test for continuous variables. The SPSS 12.0 were used for statistical calculations.

## 3. Results

During the study period, 156 patients required one type of TAC. There were no significant differences among study groups regarding age and indications for TAC (*P* > 0.05) (Table 1). Mean number of operations required in Bogota Bag (BB) group was 3.04 as compared to 1.96 in vacuum-assisted closure (VAC, KCI, San Antonio, TX, USA) group (*P* = 0.006). Survival was significantly increased in the VAC group (*P* < 0.001). The difference in primary closure rates did not reach statistical significance (25% versus 55%; *P* = 0.074). Complications were observed less frequently in the VAC group (*P* = 0.047). The median time for fascial closure was 24 days in the BB group, as opposed to 6 days in the VAC group (Table 2). 

## 4. Discussion

In the present study, VAC was found superior to BB in the management of open abdomen in terms of number of operations, survival, primary closure, complications, and mean closure time. Both groups were similar regarding age and indications for TAC.

The primary closure rates were 25% in the VAC and 55% in the BB groups, without reaching statistical significance. Fascial closure was previously evaluated in a prospective trial, and a 88% closure rate after damage control surgery using VAC was reported [6]. The complication rates, however, were significantly lower in the VAC group (50% versus 20%; *P* = 0.047). The negative pressure therapy has also been translated into practice in intra-abdominal hypertension and intra-abdominal sepsis with successful outcomes [7, 8].

In our setting we were confronted predominantly with an intra-abdominal sepsis patient population requiring management with open abdomen and observed a closure rate of 55% with VAC. The delayed primary closure rates were reported as high as 90% using VAC therapy in a predominantly trauma population after damage control laparotomy [6, 9]. In a recent study, >4 reoperations were shown to be associated with failed delayed primary closure [10]. Nevertheless, the increased survival in this cohort renders the VAC therapy an excellent tool to control intra-abdominal sepsis.

In the present series, survival was significantly increased when VAC was applied (16.3% versus 69.0%, *P* < 0.001). The success for delayed primary closure is reported to be decreased, when VAC therapy is applied in severe sepsis to control the infection. However, the method was shown to be successful in decreasing mortality in this subset of patients [11, 12]. 

## 5. Conclusion

In our experience, VAC therapy resulted in fewer reoperations, increased survival, and decreased complications. It is the current treatment of choice in the management of open abdomen, both traumatic and nontraumatic.

## Figures and Tables

**Table 1:** Characteristics.

	Bogota bag (*n* = 98)	VAC (*n* = 58)	*P* value
Mean Age (years ± SD)	50.9 ± 12.4	51.1 ± 13.4	0.920
Hollow viscus perforation	40 (40.8%)	23 (39.7%)	1.000
Anastomotic leakage	24 (24.5%)	18 (31.0%)	0.455
Abdominal compartment syndrome	21 (21.4%)	17 (29.3%)	0.335
Damage control surgery	13 (13.3%)	11 (19.0%)	0.365

**Table 2:** Outcomes.

	Bogota bag (*n* = 98)	VAC (*n* = 58)	*P* value
Mean number of operation	3.04 (1–6)	1.96 (1–3)	0.006
Survival	16 (%16.3)	40 (69.0%)	<0.001
Primary closure	4 (25%)	22 (55%)	0.074
Complications	8 (50%)	8 (20%)	0.047
Mean closure (days ± SD)	21 (±12)	6 (±3)	<0.001
